# Upregulation of TLR1, TLR2, TLR4, and IRAK-2 Expression During ML-1 Cell Differentiation to Macrophages: Role in the Potentiation of Cellular Responses to LPS and LTA

**DOI:** 10.5402/2012/641246

**Published:** 2012-05-21

**Authors:** Kassim Traore, Barry Zirkin, Rajesh K. Thimmulappa, Shyam Biswal, Michael A. Trush

**Affiliations:** ^1^Department of Chemistry Geology & Physics, Elizabeth City State University, Elizabeth City, NC 27909, USA; ^2^Department of Biochemistry and Molecular Biology, Johns Hopkins University, Baltimore, MD 21205, USA; ^3^Department of Environmental Health Sciences, Johns Hopkins University, Baltimore, MD 21205, USA

## Abstract

12-O-tetradecanoylphorbol 13-acetate (TPA) induces the differentiation of human myeloid ML-1 cells to macrophages. In the current study, the expression, responsiveness, and regulation of toll-like receptors (TLRs) in TPA-induced ML-1-derived macrophages were investigated. We have found that TPA-induced differentiation of ML-1 cells was accompanied by the upregulation of TLR1, TLR2, TLR4, and CD14 expression at both the mRNA and protein levels. Interestingly, TLR1 and TLR4 protein expression on ML-1 cells could be blocked by pretreatment with U0126, suggesting the role of an Erk1/2-induced differentiation signal in this process. In addition, the expression of IRAK-2, a key member of the TLR/IRAK-2/NF-**κ**B-dependent signaling cascade was also induced in response to TPA. Accordingly, we demonstrated an increased cellular release of inflammatory cytokines (TNF-**α** and various interleukins) upon stimulation with LPS and LTA ligands for TLR4 and TLR2, respectively. Furthermore, using luminol-dependent chemiluminescence, addition of LPS and LTA induces a sustained DPI-inhibitable generation of reactive oxygen species (ROS) by the differentiated ML-1 cells. Together, these data suggest that the increase in the responsiveness of TPA-treated ML-1 cells to LPS and LTA occurs in response to the upregulation of their respective receptors as well as an induction of the IRAK-2 gene expression.

## 1. Introduction

Humans are confronted with a number of microorganisms, viruses, and parasites that require continued surveillance and defense against [1, 2]. polymorphonuclear leukocytes (PMNs), monocytes, and tissue macrophages are important cells in the innate immune response to microorganisms and parasites [1, 3]. All derived from the same myeloid progenitor cell in the bone marrow [2]. Blood monocytes are an intermediate stage of development which then further differentiates in tissues to various macrophage populations. Tissue macrophages, as well as, other cells of the innate immune system, are critical for these surveillance and defense activities [3]. Upon stimulation by microbial particles or other endogenous factors such as cytokines, macrophages can de novo synthesize and release a large variety of cytokines (IL-1, IL-6, IL-8, IL-10, IL-12, TNF*α*, IFN*α*, IFN*γ*, MCP-l, MCP-3, MIF, M-CSF, G-CSF, GM-CSF, MIP-l, MIP-2, LIF, OSM, and TGF-*β*). Some cytokines can stimulate the production of cytokines by macrophages (IL-3, GM-CSF, IFN*γ*), while others can suppress it (IL-4, IL-10, IL-13, TGF*β*). In addition, in many organs, tissue macrophages serve as important regulatory cells [4], through their synthesis and secretion of a spectrum of cytokines and growth factors [5–7].

Toll-like receptors (TLRs) of mammalian innate immune defense systems play a crucial role in the initiation of adaptive immune responses to bacteria invasion [8–11]. A critical feature of macrophages required for specific recognition and response includes the expression of optimum levels of different TLRs [8–11]. Upon the interaction with specific pathogen-associated molecular patterns (PAMPs), TLRs trigger the signal transduction cascades that lead to cellular responses including the engulfment of invasive microbes, induction of inflammatory cytokines, and generation of ROS to kill the invasive pathogens. Several mammalian TLRs genes have been identified, and at least ten human TLRs have been cloned. TLR1, predominantly distributed in monocytes/macrophages and PMNs, is implicated with TRL2 in the recognition of the native configuration of mycobacterial lipoprotein and several other lipopeptides [10, 12]. TLR2 recognizes Gram-positive bacteria-derived Lipoteichoic acid (LTA). TLR4 and CD14, on the other hand, are required for the recognition of the Gram-negative bacteria-derived lipopolysaccharide (LPS). During sepsis, the binding of LPS to extracellular domain of TLR4 receptors on phagocytic cells, macrophages, and neutrophils elicits activation of the TLR4/IRAK-2/NF-*κ*B complex to signal production and release of proinflammatory mediators (cytokines) phagocytic cells [13]. Although TLRs play an important role in the adequate response of immune cells, the deregulation of their expression and function causes defective immune responses [14–16] and the occurrence of chronic diseases [17–19].

To understand the molecular mechanisms underlying macrophage differentiation, investigators have turned to human myeloid cell lines such as HL-60, U937, THP-1, and ML-1 [20–23]. *In vitro*, these myeloid cell lines continuously proliferate in suspension culture and can be induced to differentiate into macrophages by 12-O-tetradecanoylphorbol-13-acetate (TPA) or 1,25-dihydroxyvitamin D3 [24]. These cell lines have been widely used as cell models for studying the molecular and cellular aspects of myeloid differentiation. ML-1 cells were originally isolated from a patient with acute myeloblastic leukemia [25, 26]. These cells readily differentiate into macrophages in response to a low concentration of TPA (0.3 ng/mL or 0.5 nM) [25, 26]. In this process, the differentiation signal induced by TPA is mediated via a redox-regulated extracellular receptor kinase (Erk1/2) [27]. Differentiated ML-1 cells demonstrate a number of biochemical and functional endpoint indicators of macrophage differentiation including: nonspecific esterase (NSE) phagocytosis, plasma membrane NADPH oxidase and accompanying superoxide generation, and cell surface markers [28–31]. In the present study, we have characterized the temporal upregulation of TLR1, TLR2, TLR4, and IRAK2 expression during the differentiation of ML-1 cells leading to increased responsiveness to LPS and LTA. Our data also show that the increased expression of TLR1 and TLR4 induced by TPA is at least in part mediated through activation of ERKs.

## 2. Material and Methods

### 2.1. Antibodies and Reagents

Lipoteichoic acid (LTA) (major constituent of the Gram-positive bacteria cell wall) and lipopolysaccharide (LPS) (Escherichia coli) were purchased from Sigma-Aldrich. The human cytokines IL-1 alpha, IL-1 beta, IL-6, IL-8, IL-10, IL-12(p40), GMCSF, IFN-gamma, TNF-*α* and IP-10 cytokine multiplex immunoassay reagents were obtained from Upstate Biotechnology, Lake Placid, NY, USA. The Alexa Fluor 488-labeled mouse anti-human Abs against TLR1, TLR4, CD14, and Alexa Fluor 488-labeled isotype controls (mouse IgG1) were obtained from Molecular Probes.

### 2.2. Cell Culture

Human myeloid leukemia ML-1 cells, originally isolated from a patient with acute myeloblastic leukemia, were kindly provided by Dr. Ruth Craig, Dartmouth School of Medicine, NH. All cell culture media and supplements were obtained from Invitrogen (Carlsband, CA). Cells were cultured in RPMI 1640 Medium supplemented with 10% heat-inactivated fetal bovine serum, 2 mM L-glutamine, antibiotics (50 U/mL penicillin and 50 *μ*g/mL streptomycin), and maintained at 37°C in a humidified 5% CO_2_ atmosphere as previously described [25, 26, 32]. Experiments were routinely carried out using cells in the log phase of growth. The differentiation of ML-1 cells to macrophages entails a 6-day protocol: three days exposure to TPA (5 nM) and followed by three days of culture in the absence of TPA. Both the concentration of TPA used and the time of its presence are critical for optimal macrophage differentiation, particularly mitochondrial maturation.

TPA (Sigma Chemical Co., St. Louis, MO) was dissolved in DMSO to obtain a 100 *μ*M stock solution and further diluted before use. For all experiments, cells were cultured at an initial density of 5 × 10^5^ cells/mL. For negative controls, the cells were incubated in the absence of TPA, in medium containing an equivalent 1% (v/v) DMSO. Viability was determined by hemocytometer counts of trypan blue-impermeable cells.

### 2.3. Microarray Analysis

ML-1 cells were grown at density of 10^5^ cells/mL in a 150 mL dish and treated with vehicle (DMSO) or 5 nM TPA (*n* = 3). Total RNA was extracted using Trizol reagent and purified with the Qiagen RNAeasy minikit. The purified RNA was subjected to Affymetrix oligonucleotide microarray analysis using Human Genome U133 2.0 Plus Array Chip. Six replicates, including three controls and three TPA treated ML-1 cells samples, were examined.

### 2.4. Determination of TLR-1, TLR-4, and CD14 by FACS Analysis

The levels of TLR1, TLR4, and CD14 were determined using FACS-Scan analysis. Cells (1 × 10^6^) resuspended in PBS containing 0.1% sodium azide and 5% FBS were incubated on ice for 30 min and then incubated with (Alexa-fluor 488) conjugated anti-TLR1, TLR4, or CD14 antibodies for 1 hour. The cells were washed twice, fixed in 2% formaldehyde in PBS, and analyzed by FACS-Scan analysis. Negative controls were stained with isotype-matched (Alexa-Fluor 488)-conjugated IgG and compensation was adjusted using the single-stained cell samples. The fluorescence intensities were determined using Cellquest software (Becton Dickinson, Bedford, MA).

### 2.5. Measurement of Inflammatory Mediators

 Selected panel of inflammatory mediators (IL-1 alpha, IL-1 beta, IL-6, IL-8, IL-10, IL-12(p40), GMCSF, IFN-gamma, TNF-a, and IP-10) in cell culture medium were analyzed following stimulation of ML-1 cells or differentiated ML-1 cells (dML-1) with LPS or LTA for 16 hours, using cytokine multiplex immunoassay reagents (Upstate Biotechnology, Lake Placid, New York, United States) analyzed by Luminex 100 (Luminex Corporation, Austin, Texas, United States).

### 2.6. Measurement of ROS Generation by Luminol-Derived Chemiluminescence

The generation of cellular ROS, in particularextracellular H_2_O_2,_ was assessed using horseradish peroxidase-dependent luminol-derived chemiluminescence(CL) as previously described [33]. One million cells were incubated with luminol (10 *μ*M) and horseradish peroxidase (10 *μ*g/mL) in complete PBS, 500 *μ*L (PBS with 0.5 mM MgCl_2, _0.7 mM CaCl_2_, 0.1% glucose). Cellular CL was measured continuously in a Berthold LB9505 luminometer (Pforzheim, Germany) for 1.5 hours.

## 3. Results

### 3.1. TPA Induces TLR1, TLR2, TLR4, and IRAK-2 Gene Expression in ML-1 Cells

**Figure 1:**
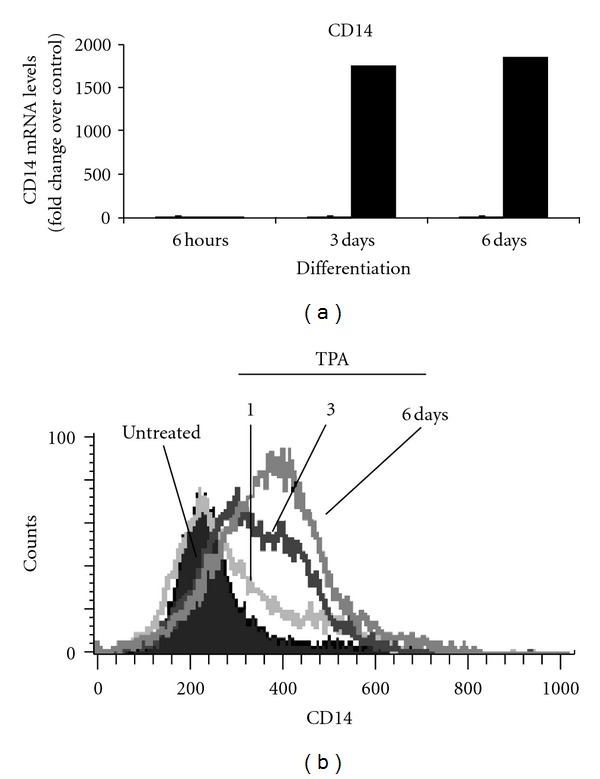
Human myeloid ML-1 cell treatment with TPA is associated with the induction macrophages specific protein CD14 expression at both mRNA and protein levels. ML-1 cells were incubated in the presence or in the absence of 5 nM TPA for the indicated times. (a) CD14 mRNA levels were determined by examination of microarray analysis data (see Section 2). (b) The levels of CD14 protein expression on TPA-treated ML-1 cells were determined by FACS-Scan analysis using Alexa-Fluor 488 labeled anti-human CD14 antibodies. Negative controls were stained with isotype-matched- (Alexa-Fluor 488) conjugated IgG, and compensation was adjusted using the single-stained cell samples. The fluorescence intensities were determined using Cellquest software (Becton Dickinson, Bedford, MA). Data were analyzed by ModFILT statistical software. The figure depicts representative results from one of three replicate experiments.

**Figure 2:**
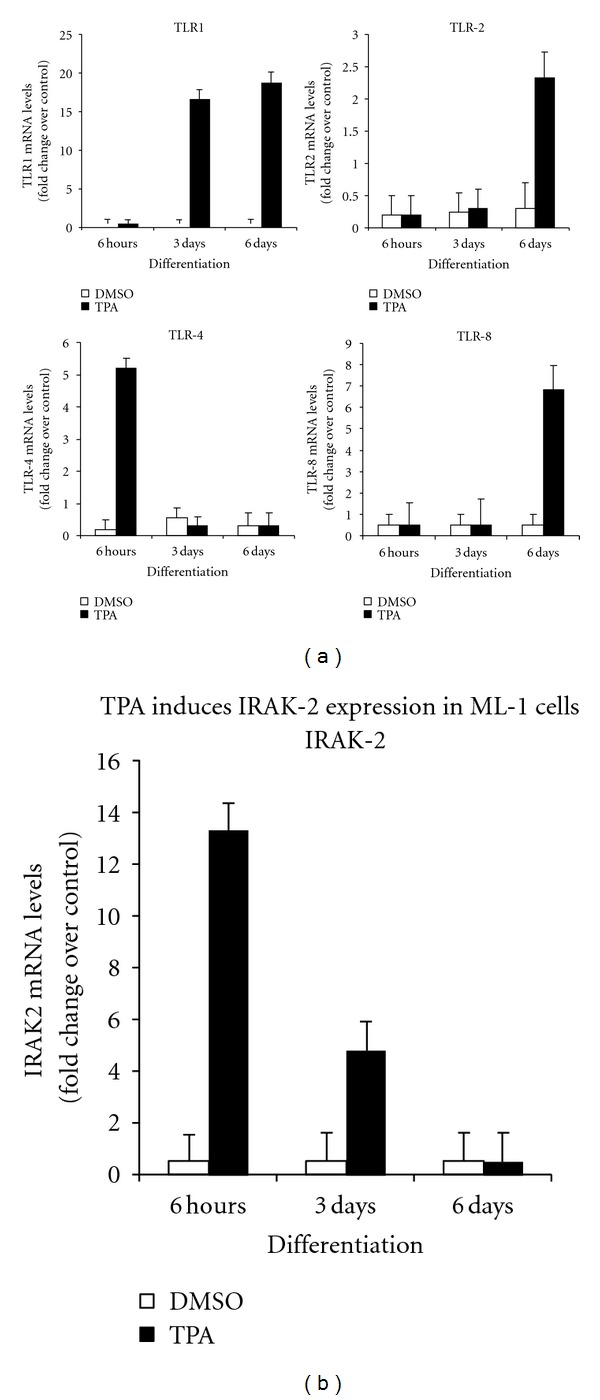
Expression of TLR mRNA expression in ML-1 cells. Six million cells were incubated in the presence TPA of (5 nM) or vehicle (DMSO) for indicated times. Total RNAs were extracted from cell samples using Trizol reagents, purified with the Qiagen RNAeasy mini kit and analyzed by microarray analysis using human Genome U133 2.0 PlusArray Chip. Six replicates including three controls (DMSO-treated) and three TPA-treated ML-1 cells samples were examined. The levels of (a) TLR1, TLR2, TLR4 and TLR8 and (b) IRAK-2 mRNAs were determined by examination of the microarray analysis data.

In our previous studies, we have demonstrated that human myeloid ML-1 cells differentiate to macrophages upon stimulation with the phorbol ester TPA [34]. To further confirm that TPA-treated ML-1 cells exhibit macrophage characteristics, CD14 gene expression, a key macrophage marker, was determined at the mRNA and protein level. Examination of the microarray analysis (Figure 1(a)), and flow cytometry analysis (Figure 1(b)) revealed a strong induction of CD14 gene expression upon stimulation with TPA and in a time-dependent fashion. Toll-like receptors (TLRs) of mammalian innate immune defense systems play a crucial role in the initiation of adaptive immune responses to bacteria invasion [8–11]. Accordingly, we assessed the temporal expression of different TLR gene products in response to TPA-induced macrophage differentiation of Ml-1 cells. Analysis of microarray data revealed significant increases in the mRNA levels of TLR1, TLR2, TLR4, and TLR8 expression also that of IRAK-2 in ML1 cells compared to control (DMSO-treated cells) (Figure 2). Interestingly, only the gene expression of the TLR members indicated above were changed in response to TPA. As shown in Figure 2, the mRNA for each of the TLRs showed different expression patterns, with TLR4 showing maximum mRNA as early as 6 hours of TPA addition, after which it decreased. Interestingly, IRAK-2 mRNA exhibited the same temporal pattern as TLR4 (Figure 2(b)). The mRNA for TLR1 appeared at 3 days and remained constant. On the other hand, the mRNA for TLR2 and TLR8 was apparent at 6 days of differentiation.

**Figure 3:**
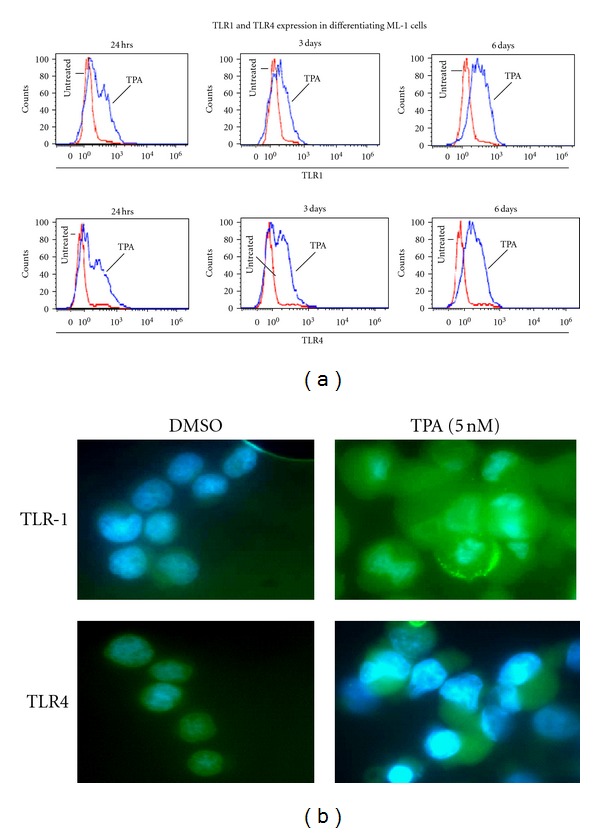
TPA induces upregulation of TLR1 and TLR4 proteins expression in human myeloid ML-1 cells. Three millions cells were incubated with 5 nM TPA for various times (24 hrs, 3 days, and 6 days). (a) Cells were then harvested, washed with PBS, fixed with formaldehyde (2%), permeabilized with ice methanol, and stained using (Alexa-Fluor-488-) labeled anti-human TLR1 or TLR4 antibodies. The levels of TLR1 or TLR4 protein expression in TPA-treated ML-1 cells were determined by FACS-Scan analysis. Negative controls were stained with isotype-matched- (Alexa-Fluor 488) conjugated IgG and compensation was adjusted using the single-stained cell samples. The fluorescence intensities were determined using Cellquest software (Becton Dickinson, Bedford, MA). Data were analyzed by ModFILT statistical software. The figure depicts representative results from one of three replicate experiments. (b) After transfer onto microscope slides, cells were washed with PBS, fixed with formaldehyde (2%), permeabilizes with PBS containing 0.25% Triton X-100, and stained using (Alexa-Fluor 488 labeled anti-human TLR1 or TLR4 antibodies). The levels of TLR1 or TLR4 protein expression were examined using fluorescent microscope.

**Figure 4:**
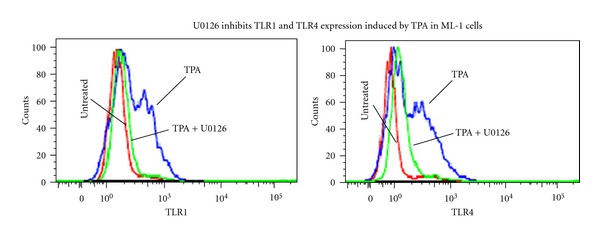
To determine the role of mitogen-activated protein kinase signal in TPA-induced TLR1 and TLR4 expression in ML-1 cells. Three millions cells were pretreated with 10 *μ*M U0126 for 10 minutes followed by incubation with 5 nM TPA for 24 hours. Cells were then harvested, washed, fixed, permeabilizes, and stained using (Alexa-Fluor-488-) labeled anti-human TLR1 or TLR4 antibodies. The levels of TLR1 or TLR4 protein expression in TPA-treated ML-1 cells were determined by FACS-Scan analysis. Negative controls were stained with isotype-matched- (Alexa-Fluor 488) conjugated IgG and compensation was adjusted using the single-stained cell samples. The fluorescence intensities were determined using Cellquest software (Becton Dickinson, Bedford, MA). Data were analyzed by ModFILT statistical software. The figure depicts representative results from one of three replicate experiments.

 Flow cytometry analysis confirmed enhanced TLR1 and TLR4 protein expression by ML-1cells (Figure 3(a)). Immunocytochemistry data revealed a basal level of expression of TLR4 but not TLR1 in the undifferentiated ML-1 cells (Figure 3(b)), which increased with macrophage differentiation. In our previous studies [23, 27, 35], we reported that the mitogen-activated protein kinase (MAPK) cascade plays a crucial role in the initiation of the macrophage differentiation signal induced by TPA. Using U0126, a MEK inhibitor, we examined whether MAPK-derived differentiation signal was required for TLR-1 and TLR-4 expression-induced by TPA in ML-1 cells. Pretreatment with 10 *μ*M U0126 effectively blocked both TLR1 and TLR4 protein expression induced by TPA in ML-1 cells (Figure 4). Since ML-1 cells already expressed a basal level of TLR4 protein, the observed increase in LPS and LTA response may be due in part to the upregulation of IRAK-2 gene expression in response to TPA. Together, these observations suggested that TLR1, TLR2 TLR4, and CD14 and IRAK-2 gene expression is induced in ML-1 cells upon stimulation with TPA. This observation is supported by a previous report that TPA induces TLR2 expression in U937 cells [36].

### 3.2. Differentiated Human Myeloid ML-1 Cells Exhibit Production of Inflammatory Cytokines in Response to LPS and LTA

**Figure 5:**
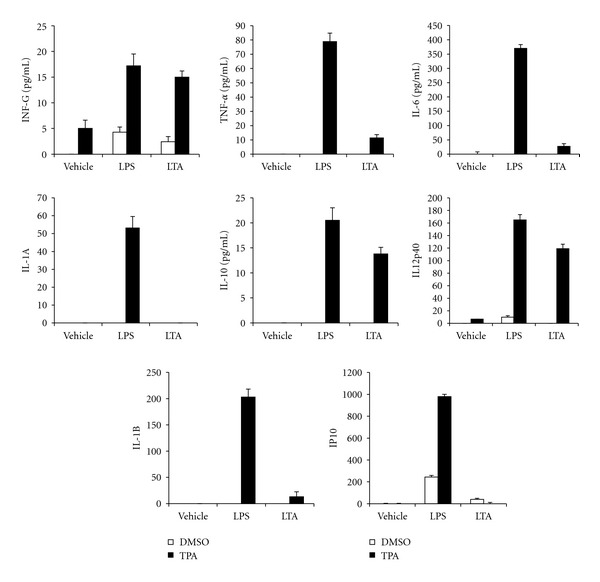
Enhanced LPS and LTA responsiveness in ML-1 cells. One millions cells were incubated for 3 days with medium alone or medium containing 5 nM TPA. Cells were washed after 3 days with PBS to remove TPA, followed by incubation with medium alone for additional three days to complete the differentiation process. Six days differentiated cells were gently washed with PBS and incubated in the absence or in the presence of 100 ng/mL LPS or 10 ng/mL LTA for 16 h. The culture medium was collected, and the levels of selected inflammatory mediators proteins (IL-1 a, IL-1 b, IL-6, IL-8, IL-10, IL-12(p40), GMCSF, IFN-gamma, TNF-a, and IP-10) in cell culture medium were determined using cytokine multiplex immunoassay reagents (Upstate Biotechnology, Lake Placid, New York, United States) analyzed by Luminex 100 (Luminex Corporation, Austin, TX, USA). Data are mean −SE of four separate experiments.

To determine whether TPA-differentiated ML-1 cells exhibit macrophage-like responses following stimulation with bacterial particles LPS and LTA, cytokine multiplex immunoassay was used to assess the levels of selected cytokine production and release. Incubation with LTA (10 *μ*g/mL) and LPS (100 ng/mL) resulted in robust production of various cytokines by the differentiated ML-1cells (Figure 5). However, there were several differences noted between LPS and LTA. For example, LPS either induced IL-1 alpha and beta, IL-6, TNF alpha and IP-10, or to a much greater extent than LTA. The presence of IL-10, IL-12p40, and interferon gamma was similar in response to LPS and LTA. These observations support our previous findings that TPA treatment induces ML-1 cell differentiation to functional macrophages [20].

### 3.3. LPS and LTA Induces Sustained Generation of ROS in Differentiated ML-1 Cells

**Figure 6:**
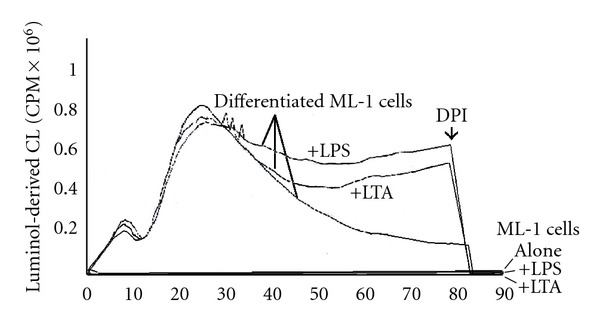
LPS and LTA induce sustained generation of reactive oxygen species (ROS) in TPA- differentiated ML-1 cells. ML-1 cells were incubated with TPA 5 nM for three days followed by incubation with growth media in absence of TPA for three days. The differentiated ML-1 cells were harvested, washed with PBS, and ROS assessed using Luminol-derived luminescence. Extracellular hydrogen peroxide produced by 1 million cells was measured in Berthold luminometer in the presence or absence of 100 ng/mL LPS or 10 ng/mL. CL is quantified as cpm x 10^6^ over the indicated time period. As indicated on the time curve, 10 *μ*M DPI was injected directly into the reaction mixture. The luminol-dependent CL of undifferentiated ML-1 cells is at the background level of the Berthold luminometer. The figure depicts results from one of three replicate experiments.

To determine whether TPA-differentiated ML-1 cells exhibit a change in ROS levels in response to LPS and LTA, luminol-derived CL was used. Horseradish peroxidase-dependent luminol-derived CL indicates the presence of extracellular hydrogen peroxide [31]. As shown in Figure 6, ML-1 cells exhibit a significant luminol-derived CL, indicative of the presence of extracellular hydrogen peroxide. We have previously determined that in unstimulated macrophages that this basal ROS is mitochondrial in origin [31]. No luminol-derived CL is observed with undifferentiated ML-1 cells. The differentiated ML-1 cells exhibited a delayed but a more sustained production of hydrogen peroxide in the presence of LPS or LTA. Addition of DPI (10 *μ*M), a potent inhibitor of NAD(P)H-dependent enzymes, rapidly suppressed the luminol-derived CL suggesting a role of NADPH oxidase and/or mitochondria complex I in the observed ROS production by ML-1 cells (Figure 6). The basal unstimulated ROS production by differentiated ML-1 cells was also blocked by DPI, as previously shown (Figure 6). Interestingly, the sustained, increased ROS with LPS or LTA treatment was not apparent until 30–40 minutes after their addition. In this regard, ROS from NADPH oxidase in alveolar macrophages is apparent almost immediately after TPA addition to activate PKC [37].

## 4. Discussion

Macrophages are key members of the innate immune system and are known to play crucial roles in initiating and maintaining the immune response [38, 39]. Our immunocytochemistry data show a basal expression of human myeloid ML-1 cells differentiate into macrophage-like cells when incubated with TPA [20]. We have demonstrated that the TPA-induced differentiation signal is mediated via a redox-mediated activation of the extracellular-regulated kinase (Erk1/2), a family member of mitogen-activated protein kinases, MAPKs, to signal cell growth arrest and cell attachment during the initiation of the differentiation process [23, 27]. In the present study, we investigated the mechanism by which TPA treatment may lead to increase in ML-1 cell response to stimulation by bacterial particle LPS and LTA. We have shown that TPA treatments result in the upregulation of TLR1, TLR2, TLR4, and CD14 in ML-1 cells, which lead in part to the potentiation of their responses to LPS and LTA. This observation correlates with a previous study by Shuto et al. in 2007, which has suggested the existence of a direct correlation between the levels of TLR2 proteins expression and the cellular response to TLR2 ligands in differentiated HL-60 cells [40]. In addition, a study by Jang et al. in 2005 has also shown an induction of TLR2 gene expression in response to TPA in U937 cells [36]. Our immunocytochemistry data show a basal expression of TLR4 but not TLR1 in undifferentiated ML-1 cells. However, upon TPA treatment, both TLR1 and TLR4 expressions were upregulated not only at the mRNA but also at the protein level.

An interesting finding is that interleukin-1 receptor-associated kinases (IRAK-2) mRNA were significantly upregulated in response to TPA treatment in ML-1 cells. The induction of IRAK-2 expression in ML-1 cells correlates with the observed increase responsiveness to TLR2 and TLR4 ligands (LPS and LTA). IRAK-2 has been implicated to participate in multiple toll-like receptor signaling pathways that lead to NF-*κ*B activation and inflammatory cytokine production [41, 42]. IRAK-2 loss-of-function mutants or knock-down of IRAK-2 expression by small interfering RNA suppresses TLR3, TLR4, and TLR8 signaling to NF-*κ*B in human cell lines, and importantly, TLR4-mediated chemokine production in primary human cells [41, 42]. Together, these observations strongly suggest that TPA treatment not only induces TLR1, TLR2, and TLR4 genes expression, but also that of the IRAK-2 in ML-1 cells. IRAK-2 participates in multiple toll-like receptor signaling pathways that lead to NF-*κ*B via activation and inflammatory cytokine production. The regulation of IRAK-2 gene expression is known to play a role in the control mechanism of inflammatory responses. Many different studies have linked IRAK-2 to a control point for TLR4-mediated signaling [41]. Since ML-1 cells express basal TLR4 level, the upregulation of IRAK-2 gene expression in response to TPA appears to be at least in part a determinant factor in the increased NF*κ*B-mediated cytokines production induced by LPS and LTA. TLRs expression on macrophages is essential for recognition and response to bacterial particle [8–11, 43]. As such, impaired expression of TLRs has been associated with defective innate immune response [14]. For example, macrophages from TLR1^−/−^ mice and TLR2^−/−^ mice were shown to be hyporesponsive to Borrelia burgdorferi outer-surface lipoprotein (OspA) vaccination [16]. Therefore, defects in the TLR1/2 signaling pathway may account for human hyporesponsiveness to OspA vaccination. Interestingly, recent studies have proposed that the overexpression of TLR4 may play roles in the occurrence of inflammatory diseases [17–19]. Our suggestion that TLR1 and TLR4 expression required MAPK activation is based on (i) our previous observations that TPA induces a ROS-mediated Erk1/2 activation in ML-1 cells [23, 27] and (ii) the TLR1 and TLR4 protein expression in ML-1 induced by TPA was blocked by U0126, an agent that is known to effectively suppress MEK activity. This observation is consistent with previous reports by Jang et al. [36] that demonstrated that TPA-induced TLR2 expression in U937 cells was mediated via an Erk1/2 and ROS-independent signal mechanisms [36].

ROS generation in response to bacterial LPS and LTA is crucial for pathogen killing by immune cells [8, 44]. However, sustained production of ROS during immune responses and sepsis can cause damage to macromolecules, cell death and tissue injury [45, 46]. In TPA-differentiated ML-1 cells we have identified two sources of ROS, NAD(P)H oxidase, and the mitochondrial electron transport chain [27]. Here, we demonstrated that TPA-differentiated ML-1 cells exhibit a prolonged generation of ROS upon stimulation with LPS and LTA. Our conclusion that the mitochondrial electron transport chain and/or NADPH oxidase may be the source(s) of this ROS is based on the observation that DPI (10 *μ*M), an agent that we have shown effective in inhibiting both NADPH oxidase and mitochondria complex Iin ML-1 cells [27], immediately and completely suppressed the prolonged ROS generation induced by LPS and LTA. While DPI inhibition does not allow us to conclude the source of ROS elicited by LPS and LTA, there are several studies that have demonstrated interactions between TLR and mitochondria. For example, a recent study has shown that a subset of toll-like receptors (TLR1, TLR2, and TLR4) promote recruitment of mitochondria to macrophage phagosomes and induce the generation of mitochondrial ROS. These results implicate mROS as an important component of antibacterial responses and establish mitochondria as hubs for innate immune signaling [8]. Similarly, toll-like receptor 4 mediates ROS-mediated mitochondrial DNA damage and biogenic responses in the liver of mice exposed to heat-inactivated E. coli [47]. Collectively, the data presented in this study demonstrate that TPA-induced differentiation of ML-1 cells to macrophages is accompanied by the expression of members of the TLR/IRAK-2/NF-*κ*B complex. This leads to a robust downstream TLR/IRAK-2/NF*κ*B-mediated cytokine production and an increase in sustained cellular oxidative metabolism and ROS upon cellular interaction with bacterial LPS and LTA.

## Abbreviations

TPA, LPS, LTA, ROS: 12-tetradecanoyl-phorbol-13-acetate.
